# Pyroptosis Plays a Role in Osteoarthritis

**DOI:** 10.14336/AD.2019.1127

**Published:** 2020-10-01

**Authors:** Senbo An, Huiyu Hu, Yusheng Li, Yihe Hu

**Affiliations:** ^1^Department of Orthopedics, Xiangya Hospital, Central South University, Changsha, Hunan, China.; ^2^Department of General Surgery, Xiangya Hospital, Central South University, Changsha, Hunan, China.; ^3^National Clinical Research Center for Geriatric Disorders, Xiangya Hospital, Central South University, Changsha, Hunan, China.

**Keywords:** pyroptosis, caspase, inflammation, osteoarthritis

## Abstract

Recent studies have revealed novel forms of cell death beyond the canonical types of cellular apoptosis and necrosis, and these novel forms of cell death are induced by extreme microenvironmental factors. Pyroptosis, a type of regulated cell death, occurs when pattern recognition receptors (PRRs) induce the activation of cysteine-aspartic protease 1 (caspase-1) or caspase-11, which can trigger the release of the pyrogenic cytokines interleukin-1β (IL-1β) and IL-18. Osteoarthritis (OA), the most common joint disease worldwide, is characterized by low-grade inflammation and increased levels of cytokines, including IL-1β and IL-18. Additionally, some damaged chondrocytes associated with OA exhibit morphological changes consistent with pyroptosis, suggesting that this form of regulated cell death may contribute significantly to the pathology of OA. This review summarizes the molecular mechanisms of pyroptosis and shows the critical role of NLRP3 (NLR family, pyrin domain containing 3; NLR refers to “nucleotide-binding domain, leucine-rich repeat”) inflammasomes. We also provide evidence describing potential role of pyroptosis in OA, including the relationship with OA risk factors and the contribution to cartilage degradation, synovitis and OA pain.

## 1.Introduction

Cell death is one of the most fundamental physiological processes used by multicellular organisms to maintain homeostasis, as exemplified by tissue sculpting during embryologic development and the elimination of senescent cells. Cell death is also a characteristic of abnormal pathological responses to harmful stimuli, such as physical damage and pathogens [1]. Traditionally, the literature concerning cell death has been based on the morphology of dying cells and is dominated by apoptosis and necrosis [2]. Apoptosis, a process driven by the caspase family of enzymes, is the most studied and best characterized form of programmed cell death [3]. Morphological changes observed in apoptotic cells include DNA fragmentation, decreased cell volume and cytoplasmic and membrane vesicle formation [4]. Ultimately, apoptotic cells are cleared rapidly by adjacent phagocytes without inducing an inflammatory response. Because of these characteristics, apoptosis is considered to be “programmed” and “regulated” and is beneficial for normal embryonic development, tissue homeostasis and immune responses [5]. In contrast, necrosis is described as an uncontrolled and “accidental” process caused by exposure to overwhelming microenvironmental conditions such as extreme hyperthermia, hypothermia or radiation [6]. Necrotic cells exhibit swelling, plasma membrane rupture and increased permeability, as well as a loss of cellular contents and maintenance of DNA contents [7]. Necrosis is a strongly inflammatory process with little phagocytic involvement.

Recent studies have revealed several other types of cell death, including autophagy [8], necroptosis [9], pyroptosis [10], ferroptosis [11], mitochondrial permeability transition (MPT)-dependent necrosis [12] and parthanatos [13]. These discoveries have led to the concept that multiple forms of regulated cell death can encompass morphological and biochemical features similar to both apoptosis and necrosis [14]. Pyroptosis is a type of proinflammatory programmed cell death that is triggered by inflammasomes. This process induces cell rupture and the release of cell contents, similar to necrosis, but pyroptosis involves caspase-driven programmed cell death, which is consistent with apoptosis [15].

Osteoarthritis (OA) is the most common chronic degenerative disease worldwide. Although OA is highly prevalent [16], it mainly affects elderly people. This disease can involve nearly any joint in the human body, and the most common symptoms include joint pain and disordered articular functions [17]. The pathological changes associated with OA affect all tissues in the joint and include cartilage degeneration, subchondral sclerosis, variable degrees of synovial inflammation, osteophyte formation and hypertrophy of the whole joint capsule [18].

Inflammasomes such as NLRP3 (NLR family, pyrin domain containing 3; NLR refers to “nucleotide-binding domain, leucine-rich repeat”), which are induced by nuclear factor kappa B (NF-κB) signaling and can convert interleukin-1β (IL-1β) and IL-18 into mature proinflammatory cytokines, are considered a factor in low-grade inflammatory pathology [19]. Among these inflammasomes, which comprise NLRs (NLRP1, NLRP3, and NLRC4), absent in melanoma 2 (AIM2), IFN-inducible protein 16 (IFI16) and pyrin, the NLRP3 inflammasome is the best characterized. This complex comprises NLRP3, apoptosis-related speck-like protein containing (ASC) a caspase recruitment domain (CARD) and procaspase-1 [20], suggesting that pyroptosis may be closely involved in the pathological changes associated with OA. Furthermore, the frequency of OA pain was indicated to be mediated by aberrantly overexpressed cytokines and to be associated strongly with inflammatory severity, especially in soft tissues such as the synovium, which could lead to hypersensitivity with exaggerated pain (hyperalgesia) in response to noxious stimuli or innocuous stimuli that are perceived as painful (allodynia) [21]. This indicates that pyroptosis may contribute to OA pain through releasing related cytokines [22]. In this review, we aimed to identify the possible mechanism by which pyroptosis contributes to OA progression and pain. Because no efficient treatment option is currently available for OA, an understanding of pyroptosis and related signaling factors might be useful in clinical decision-making.

## 2.Pyroptosis

Classic apoptosis is a caspase-dependent process of programmed cell death. An alternative form of cell death involving caspase-dependent programmed cell death combined with proinflammatory changes has also been identified [23]. This process was termed “pyroptosis” [24] after the identification of macrophages infected with *Salmonella* or *Shigella* spp. The term for this type of proinflammatory programmed cell death was derived from the Greek roots *pyro*, meaning “fire” or “fever,” and *ptosis*, which means “falling” [25]. In contrast to other forms of programmed cell death, pyroptosis is closely related to inflammation. Morphologically, pyroptosis involves the cleavage of procaspase-1 to yield active caspase-1. This activated enzyme forms pores in the plasma membrane, which enables the equalization of the ionic gradient between the intracellular and extracellular environments. Consequently, water flows into the cell, causing swelling and lysis [26]. Along with the increase in cell size and formation of a balloon-like surrounding vesicle, the nucleus condenses and becomes rounded [27, 28]. Although DNA fragmentation consistent with apoptosis is observed, nuclear integrity is maintained in pyroptosis [29, 30]. Therefore, the morphological changes associated with pyroptosis are distinct from those associated with apoptosis, and the former process can be described as having a combination of apoptotic and necrotic features.

## 3.Molecular mechanisms of pyroptosis

Inflammasomes are multimeric protein complexes that assemble in the cytosol and act as platforms for caspase activation. These complexes play vital roles in the initiation of inflammation. Caspases are cysteine proteases that initiate or execute cellular programs. These proteases can induce inflammation or cell death, depending on their function [31] and are thus categorized as proinflammatory or proapoptotic. Proinflammatory caspases include caspases-1, -11 and -12 in mice and caspases-1, -4, and -5 in humans [32]. Of these, caspase-1, the most fully characterized, is regulated closely by the inflammasome to process cytokines [33]. Activated caspase-1 is required for proteolytic processing and for release of the cytokines IL-1β and IL-18 [34]. Caspases may be activated in response to various stimuli. Upon damage induced by endogenous stress or microbial infection, caspases release either danger-associated molecular patterns (DAMPs) or pathogen-associated molecular patterns (PAMPs) that trigger pattern recognition receptors (PRRs) [35]. PRRs can be divided into two categories based on their subcellular localization. Transmembrane PRRs include Toll-like receptors (TLRs) and C-type lectins (CTLs), while intracellular PRRs include the RIG-I-like receptor (RLR), AIM2-like receptor (ALR) and nucleotide-binding domain and leucine-rich repeat-containing (NLR) proteins [36]. The pathways associated with pyroptosis are defined by the triggering of different caspases. The canonical inflammasome pathway is triggered by activated caspase-1, while the noncanonical inflammasome pathway is triggered by activated caspase-11 (in mice) or caspase-4/5 (in humans) [37]. In the canonical inflammasome pathway, assembly of the NLRP3 inflammasome in response to PAMPs or DAMPs can lead to caspase-1-dependent release of proinflammatory cytokines and gasdermin D (GSDMD)-mediated pyroptotic cell death. With few exceptions, NLRP3 inflammasome activation is considered to have two steps: priming and activation [38]. The priming step activates the inflammatory process in cells and upregulates the expression of inflammasome components upon increased transcriptional activity of nuclear factor-κB (NF-κB) [39, 40], upregulating the expression of inflammasome components, including NLRP3, caspase-1 and pro-IL-1β. The following step is activation of NLRP3 by a variety of upstream stimuli induced by numerous PAMPs or DAMPs, including K^+^efflux[41], Ca^2+^ flux [42], lysosomal disruption [43], mitochondrial dysfunction [44], metabolic changes [45] and trans- Golgi disassembly [46]. NLRP3 then interacts with NIMA (never in mitosis gene a)-related kinase 7 (NEK7) and forms a complex with ASC and caspase-1 (the NLRP3 inflammasome). The complex cleaves GSDMD to form GSDMD-N pores into the membrane, which induces pyroptosis. The complex also enhances the processing of inactive IL-1β and IL-18 into mature inflammatory cytokines, which can in turn promote the transcriptional activities of NF-κB, activator protein 1 (AP1) and interferon regulatory factors (IRFs) [40, 47].

Noncanonical inflammasomes detect intracellular bacteria and bacterial lipopolysaccharide (LPS) molecules in infected cells [48] and consequently activate caspase-11 (in mice) or caspase-4/5 (in humans). Subsequently, caspase-11 directly triggers pyroptosis. Once activated, these caspases cleave the cellular protein GSDMD, which inserts into the cell membrane lipid bilayer via its N-terminal domain. This process enables the formation of pores that induce osmotic cell lysis [49, 50]. A previous study proved that caspase-11 also indirectly processes IL-1β via the caspase-1 pathway [37], which suggests crosstalk between different caspases during the pyroptosis.

## 4.Pyroptosis in different diseases

Several studies have investigated the roles of pyroptosis in different diseases, particularly cardiovascular diseases[51]. Existing evidence has revealed significant increases in caspase-1 expression in vulnerable plaques and ruptured lesions associated with acute coronary events in atherosclerosis [52]. Additionally, monocyte/ macrophage pyroptosis may also induce an amplified inflammatory response and promote plaque rupture and thrombosis, which can induce acute cardiovascular events [53]. Moreover, vascular smooth muscle cell pyroptosis may suppress the healing of vascular injuries and reduce the stability of atherosclerotic plaques [54, 55]. Cardiomyocyte and cardiac fibroblast pyroptosis is also involved in diabetic cardiomyopathy [56], cardiac hypertrophy [57] and ischemic heart disease [58].

Existing evidence suggests that inflammasomes such as NLRP3 can be activated by fatty acids and high glucose levels, leading to chronic intestinal inflammation [59] and colorectal cancer [60]. These findings indicate that pyroptosis plays a significant role in digestive tract inflammation as well as in obesity-associated carcinogenesis. Pyroptosis, a highly inflammatory mode of cell death, can also trigger autoimmune diseases such as systemic lupus erythematosus (SLE) [61]. LPS from gram-negative bacterial infection induce pyroptosis by activating caspase-4/5/11, contributing to infectious diseases such as sepsis [25]. Pyroptosis also plays a role in inflammatory joint diseases such as rheumatoid arthritis (RA) [62] and OA [19].

## 5.Osteoarthritis

As noted previously, OA is the most common degenerative joint disorder and can affect nearly any small or large joint in the body [63]. OA is now considered to have complex etiology, and these multiple factors can lead to similar outcomes of joint destruction and clinical features [64]. In a study of OA patients older than 50 years of age in North America and Europe, approximately 60%, 33% and 5% reported involvement of joints of the hand, knee and hip, respectively [65]. An age older than 40 years is associated with a greater likelihood of developing OA. Moreover, OA is more frequent in women than in men at any age older than 50 years (i.e., postmenopausal women), and this sex difference mainly involves the hand (e.g., distal interphalangeal) and knee joints [66]. The risk factors for OA include factors related to individual susceptibility, as well as those affecting biomechanical joint stability [67]. Obesity may also be a risk factor for OA, as it increases stress on weight-bearing joints such as the hips and knees. Additionally, fat cells produce potentially harmful inflammatory proteins that may target the joints. The genetics underlying OA are complex but highly significant and may be related to changes in important molecular pathways. The genes encoding inflammatory and catabolism-related proteins are upregulated during OA, primarily through signal transduction involving nuclear factor-κB (NF-κB), mitogen- activated protein kinase, and other inflammation- and stress-induced pathways [63, 68]. Joint injuries, such as those involving the ligaments and meniscus, can increase the fragility of the joint and increase the risk of osteoarthritis. Even injuries that occurred many years ago can increase the risk of OA. Moreover, joint malformation or cartilage defects can increase the risk of OA [69-71]. The symptoms of OA include pain, transient morning stiffness and a grating sensation when walking. End-stage OA is characterized by instability and physical disability, which have tremendous negative effects on the quality of life [72]. The lack of efficient and curative treatment options for OA and the ever-evolving pathophysiological and risk factors present significant challenges.

Because its exact pathogenesis has not been fully revealed, OA is characterized as a whole-joint disease associated with pathological changes in all involved tissues. The pathology of OA includes the progressive loss and destruction of cartilage, sclerosis of the subchondral bone, formation of osteophytes, variable degrees of synovial inflammation, degeneration of the ligaments and meniscus and hypertrophy of the whole joint capsule[73, 74]. Initially, the risk factors that cause joint instability, such as ligament injury and excessive body weight, induce the excessive secretion of transforming growth factor β1 (TGF-β1) in the subchondral bone. An excess of this cytokine leads to the uncoupling of bone formation and resorption, which is accompanied by angiogenesis, sensory innervation [21], bone cavity formation and sclerosis [75]. Articular cartilage degeneration is the primary concern in OA [76]. Normally, in an environment with low oxygen, chondrocytes exhibit a low-grade turnover rate to maintain an anabolic-catabolic balance in the cartilage [77]. In OA, however, the expression of genes that encode proteins associated with inflammatory and catabolic responses is upregulated primarily via signal transduction pathways involving NF-κB, mitogen-activated protein kinase and other factors that are activated by inflammation. This transcriptional activity increases the production of primary inflammatory cytokines, such as IL-1β and tumor necrosis factor α (TNF-α), which play critical roles in OA pathology. IL-1β and TNF-α stimulate chondrocytes to release cartilage-degrading enzymes such as metalloproteinases 1, 3 and 13 (MMP1, MMP3 and MMP13) and a disintegrin and metalloproteinase with thrombospondin motifs 4 (ADAMTS4) and ADAMTS5 [78, 79]. These enzymes digest aggrecans and type II collagen to degrade the cartilage matrix. IL-1β and TNF-α also contribute to inflammatory chondrocyte death [80]. Together, these processes lead to a gradual loss of cartilage and the formation of osteochondral fissures, which allow the formation of new endochondral bone accompanied by angiogenesis and sensory innervation [81]. These processes may be closely associated with OA pain. During the late stage of OA, patients lose joint function and present with an immensely narrowed joint space containing little remaining cartilage, especially in the knee and hip joints.

The synovium is an important source of nutrients for cartilaginous tissue, which is avascular. Therefore, synovitis, or inflammation of the synovium, plays a key role in OA pathogenesis [82]. The synovium includes the synovial membrane and fluid [83]. Damaged chondrocytes and cartilage release proteinases that increase the concentrations of proinflammatory cartilage degradation products, such as DAMPs, in the synovial fluid. These products induce inflammation in the adjacent synovium [84, 85] and present feedback to the chondrocytes for further degradation. Consequently, increased amounts of matrix-degrading enzymes are secreted to deteriorate the cartilage, and reduced amounts of lubricin and hyaluronic acid (HA) are secreted to protect the joints [86].

## 6.The role of pyroptosis in OA

### 6.1*Pyroptosis is related to OA risk factors*

To date, a limited amount of literature has described the role of the NLRP3 inflammasome in OA pathogenesis. Since strong evidence shows that NLRP3 is a potential marker in other diseases such as RA [87], atherosclerosis [88], gout [89] and colorectal cancer [90], we detailed the evidence that indicates the association of NLRP3 with OA in the following sections.

Cartilage degeneration is the most well-known pathological change associated with OA. The joint tissues of patients harboring OA risk factors, such as metabolic disorders, aging, infectious joint diseases and injuries, can generate several kinds of DAMPs or PAMPs, including adipokines, microcrystals and uric acid.

Metabolic disorders contribute to OA pathogenesis. Obesity (body mass index > 30 kg/m^2^) is a significant risk factor for the onset and progression of OA [91]. Waist circumference, which is an indicator of central adiposity, is associated with increased knee cartilage defects [92], reduced knee cartilage mass and increased bone marrow lesions [93]. Adipokines, one of the obesity-related metabolic factors, have been shown to induce cartilage degradation [64]. Leptin, an adipokine, has a detrimental effect on chondrocyte proliferation by increasing IL-1β and MMPs [94]. Additionally, individuals with obesity have low circulating 25-hydroxy-vitamin D (25-(OH)D) [95], which is positively associated with the development and worsening of knee OA, including cartilage loss and increased OA pain [96].

Adipose tissue macrophages (ATMs) exhibit a proinflammatory M1 phenotype under obese conditions but an M2-dominant phenotype under normal conditions. Proinflammatory ATMs produce tumor-promoting cytokines, including TNF-α, IL-1β, IL-6, IL-8, IL-18 and IL-32 [95], and therefore serve as a source of both local and systemic proinflammatory mediators [96]. Factors such as oxidized low-density lipoprotein (LDL) and cholesterol can activate the NLRP3 inflammasome. Vandanmagsar *et al* [97] found that ablation of NLRP3 in mice prevented obesity-induced inflammasome activation in fat deposits. In other words, obesity promotes pyroptosis by enabling the release of the NLRP3 inflammasome, which may be related to OA progression. Furthermore, humans with obesity have elevated blood LPS levels. LPS activates caspase-4/5/11, which initiates pyroptosis via the noncanonical inflammasome pathway [99] at both the systemic and tissue levels, leading to increased inflammatory chondrocyte death and loss of cartilage.

Microcrystals, including basic calcium phosphate (BCP), calcium pyrophosphate and uric acid [98], are considered “danger signals” and are thought to be the main DAMPs that activate inflammasomes. BCP crystals have been found in the knee and hip joints of OA patients [99], and these crystals elicit the release of IL-1β from macrophages by activating NLRP3 in a process that is dependent on K^+^ efflux, reactive oxygen species (ROS) and phagocytosis [100, 101]. This pyroptosis-related process drives the pathology of OA and induces cartilage degeneration. Previous reports[102] suggested that in synovial fibroblasts and articular chondrocytes, IL-36, an IL-1 cytokine subfamily member, stimulates the expression of inflammatory mediators and thus plays a critical role in inflammatory diseases [59].

Aging is also a significant risk factor for multiple chronic diseases, as elderly people are more vulnerable due to their decreased immune and metabolic activities. A highly critical event occurs with aging, which starts with DAMP accumulation and activation of the subsequent NLRP3 inflammasome, which is stimulated by endogenous byproducts [103] that are then recognized by PRRs in macrophages to trigger chronic, low-grade inflammation [31]. In turn, chronic inflammation plays an important role in the progression of metabolic disorders, such as obesity, gouty arthritis and atherosclerosis [104].

### 6.2*Pyroptosis contributes to cartilage degradation*

Upon stimulation by DAMPs, macrophages surrounding the cartilage undergo pyroptosis after caspase-1 activation by DAMPs or PAMPs and the release inflammasomes such as NLRP3. This increases the release of IL-1β and IL-18 on the cartilage surface, leading to increases in the concentrations of proinflammatory cytokines in chondrocytes and further promoting pyroptosis [105]. These cytokines stimulate chondrocytes to secrete catabolic enzymes that cause cartilage degradation, such as MMP13 and ADAMTS5 [106], thus leading to cartilage destruction.

### 6.3*Pyroptosis plays a key role in synovial changes in OA*

Chronic synovial inflammation is a known cause of cartilage degradation [87, 107]. In OA, IL-1β, which is involved in cartilage degradation, may be produced by synovial tissue rather than by chondrocytes [108]. Furthermore, the protein level of NLRP3 is more than five-fold higher in the synovial membranes of OA patients than in subjects with normal joints [109]. Genetic studies have proven a close association of inflammasomes with RA, which is a primary autoimmune disease of the synovium. These studies revealed the significantly upregulated expression of genes encoding NLRP3 inflammasome components, including NLRP3, ASC and caspase-1, in patients with RA [62, 110].

In patients with knee OA, the level of uric acid, a danger signal, in the synovial fluid was found to correlate with the synovial levels of IL-1β and IL-18 and the severity of OA [111]. Therefore, uric acid is a driver of OA pathogenesis and may be a marker of OA severity [112]. Chondrocytes produce increased IL-1β and IL-18, while IL-1β also independently induces the expression of IL-18, which enhances the local inflammatory activities independently in the surrounding synovial fluid [113], enhancing local inflammation during the progression of OA.

Briefly, in OA synovial macrophages, pyroptosis-associated inflammasomes, such as NLRP3, are induced by different DAMPs and released into the synovial fluid. The concentrations of these inflammasomes are elevated in the articular fluid and surrounding tissues. They increase the level of IL-1β and stimulate chondrocytes to produce more proinflammatory cytokines (e.g., IL-18) in a cascade of inflammatory responses involving macrophages and chondrocytes. Ultimately, this process leads to chondrocyte pyroptosis and cartilage degradation.

### 6.4*Pyroptosis contributes to OA pain*

Pyroptosis may also contribute to the pathological mechanism underlying pain, which is one of the most characteristic symptoms of OA. Continuous nociceptive input from the OA joint is thought to sensitize central and peripheral nerves and induce pain [114]. The majority of OA pain is caused by changes in peripheral nerves. Normally, nerve nociceptors are localized within specific tissues and can only detect sensations in a designated area. During OA progression, excess cytokines, chemokines and inflammatory factors, mechanical stimuli and enhanced innervation contribute to the expansion of nociceptive input [115]. In OA pathology, inflammasomes in pyroptotic macrophages upregulate the production of proinflammatory cytokines, such as IL-1β, IL-18 and TNF-α, which can increase nociceptive input in the joint tissue. This process is called “hyperalgesia.” Additionally, in the context of chronic inflammation associated with OA, pyroptosis may contribute to pathological changes in the whole joint, including the loss of cartilage, formation of osteochondral fissures and osteophytes and synovitis [116]. Furthermore, dense networks of perivascular sensory and sympathetic nerves may innervate the channels or extend into the synovium, ligament or meniscus [116, 117]. This abnormal pattern of innervation would surely contribute to OA pain.

**Figure 1.**
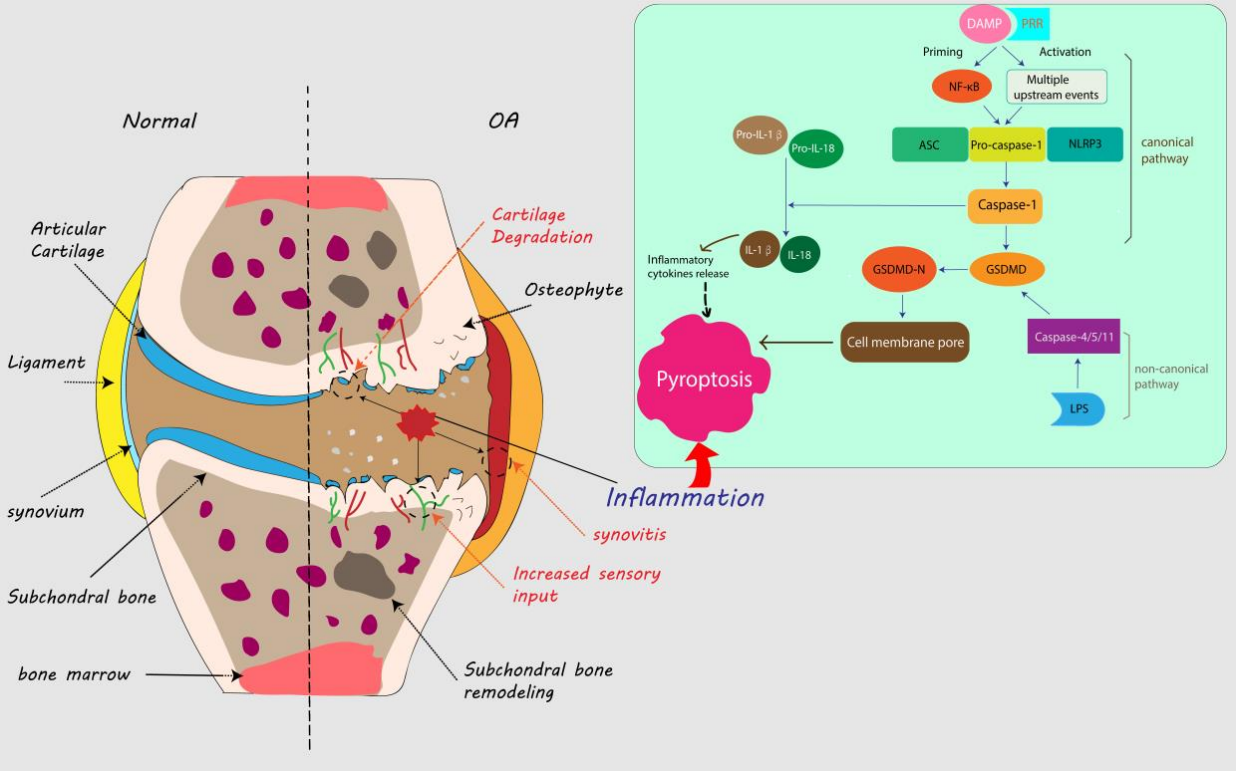
Pyroptosis in knee osteoarthritis progression. In macrophages or other cells in the synovium or synovial fluid of the knee joint, DAMPs can stimulate and combine with PRRs (e.g., TLRs) on the membrane, forming inflammasomes (ASC, pro-caspase-1 and NLRP3), which are activated by two steps: priming and activation. Inflammasomes can convert pro-caspase-1 into active caspase-1, which cleaves GSDMD and form GSDMD-N. GSDMD-N generates membrane pores and leads to cell swelling and eventual lysis. Moreover, caspase-1 induces the release of more mature proinflammatory cytokines, including IL-1β and IL-18, which can in turn lead to further inflammatory cytokine release and promote pyroptosis. This is the canonical pathway of pyroptosis. LPS can directly activate caspase-4/5 (human) or caspase-11 (mouse), which can also activate GSDMD, and this is a noncanonical pyroptosis pathway. Compared with the inflammatory activity in normal knee joints, the increased inflammatory activities caused by pyroptosis in the joint promote osteoarthritis progression, with exacerbated synovitis, cartilage degradation and increased sensory input to the central nervous system, which can generate more pain.

## 7.Conclusions and perspectives

Pyroptosis is a proinflammatory form of regulated cell death that may be strongly correlated with OA progression. The levels of DAMPs, such as microcrystals, are much higher in OA joints and can trigger activation of the NLRP3 inflammasome and caspase-1. Much higher levels of NLRP3 are found in the synovium in OA joints than in normal joints, suggesting that macrophage pyroptosis is closely related to synovial inflammation. Pyroptotic cells release IL-1β and IL-18 into the synovial fluid following the activation of caspase-1 (i.e., the canonical pyroptosis pathway). This process further increases the levels of proinflammatory cytokines in chondrocytes and enhances the inflammatory response cascade, ultimately leading to chondrocyte death and cartilage degradation. Additionally, in patients with obesity, elevated LPS levels directly trigger the noncanonical pyroptosis pathway by activating caspase-4/5. Moreover, the proinflammatory cytokines generated during pyroptosis contribute to an increase in nociceptive input in the joint, which can cause a state of hyperalgesia and OA pain. Additionally, destruction of the cartilage and other joint tissues enables excessive sensory and sympathetic innervation, which further exaggerates the sensation of pain. The molecular mechanism underlying pyroptosis is well recognized. Accordingly, the inhibition of inflammasomes such as NLRP3, caspase-1 or GSDMD may represent a novel approach to the treatment of OA.

However, our understanding of the role of pyroptosis in OA remains limited. Moreover, there were also some opposing data that do not support the role of pyroptosis in OA. Busso *et al* [118] found in a murine model that IL-1α and IL-1β were not key mediators in OA. A lack of IL-1α or NLRP3 could lead to increased cartilage erosion. Bougault *et al* [111] found that knockout of NLRP3 did not inhibit the expression of MMP3, MMP9 and MMP13 in cultured chondrocytes, and this was no changed by inhibiting caspase-1 or IL-1β. These studies all indicated that inhibiting IL-1β was not a promising therapeutic target, which was consistent with human clinical trials[119]. However, these data do not necessarily disprove the role of pyroptosis in OA. First, the animal and cartilage explant models in these studies may not accurately reflect the aspects of the disease that are mediated by pyroptosis. Second, pyroptosis may be a process that is closely related to cell death, not just inflammatory factors such as IL-1β.

Further studies should focus on the mechanism of pyroptosis in chondrocytes and other nonmacrophage cells to clarify how this process of cell death is mediated in chondrocytes within cartilaginous tissues. This knowledge would improve our understanding of the mechanism by which pyroptosis mediates OA development and could elucidate the mechanism underlying OA pathogenesis. Overall, the role of inflammasomes such as NLRP3 and their regulators in pyroptosis indicates that NLRP3 appears to be a promising biomarker for the diagnosis and monitoring of OA. Treatment targeting NLRP3 may be a potential therapeutic strategy for OA.
